# Early Predictors of Poor Neurological Outcomes in Pediatric Status Epilepticus: Initial Glasgow Coma Scale (GCS) Score, Full Outline of UnResponsiveness (FOUR) Score, and Seizure Duration

**DOI:** 10.7759/cureus.111749

**Published:** 2026-06-29

**Authors:** Anisha Pathak, Shubhra Dubey, Shalini Hajela

**Affiliations:** 1 Paediatrics, Bundelkhand Medical College, Sagar, IND; 2 Community Medicine, KD Medical College Hospital and Research Centre, Mathura, IND

**Keywords:** full outline of unresponsiveness score, glasgow coma scale(gcs), neurological outcome, seizure duration, status epilepticus (se)

## Abstract

Background and objective

Status epilepticus (SE) is one of the most critical medical emergencies that may result in significant morbidity and mortality if not addressed in a timely and effective manner. Early identification of prognostic predictors is essential for determining outcomes and guiding management. Clinical tools such as the Glasgow Coma Scale (GCS) score, Full Outline of UnResponsiveness (FOUR) score, and seizure duration are commonly used, but their independent prognostic value in pediatric SE remains uncertain. This study aimed to evaluate the association between initial GCS, FOUR score, and seizure duration and neurological outcomes in children with status epilepticus.

Methods

This prospective analytical study included 175 children aged one month to 14 years who fulfilled the inclusion criteria and were admitted to a tertiary care pediatric ICU between August 2022 and May 2024. We prospectively enrolled pediatric patients aged one month to 14 years who presented with convulsive SE (seizure duration of more than five minutes) and whose clinical scores were evaluated before sedative administration. Patients with post-cardiac arrest or trauma-related seizures were excluded. Initial GCS and FOUR scores were recorded at admission before midazolam administration. Seizure duration was documented and categorized as 5-30 minutes or more than 30 minutes. Outcomes were classified as recovery, neurological deficit, or death at discharge. Specifically, a "poor neurological outcome" was defined strictly as in-hospital mortality. A "good outcome" was defined as overall survival, which included both patients who completely recovered and those discharged with a neurological deficit. Multivariate logistic regression analysis was performed to identify independent predictors of poor outcome (defined as death).

Results

Of the 175 children, 152 (86.4%) recovered fully at discharge, eight (4.5%) developed neurological deficits, and 15 (8.5%) died. Mean GCS (10.70) and FOUR (12.20) scores were significantly lower in patients with poor outcomes compared to those who recovered (p < 0.01 for both). Seizure duration of more than 30 minutes was strongly associated with adverse outcomes (p < 0.001). On univariate analysis, a lower GCS score (p = 0.01, 95% confidence interval (CI): 0.40-0.71), a lower FOUR score (p = 0.04, 95% CI: 0.42-0.74), and prolonged seizure duration (p < 0.01, 95% CI: 9.12-77.42) independently predicted poor neurological outcome. On multivariate analysis, only seizure duration of more than 30 minutes independently predicted poor neurological outcome (adjusted odds ratio (AOR) = 12.14, 95% CI: 3.14-43.26; p < 0.001), while GCS and FOUR scores lost significance.

Conclusions

Lower GCS and FOUR scores, along with prolonged seizure duration, were significantly associated with poor neurological outcomes on univariate analysis. However, after multivariate adjustment, only seizure duration remained an independent predictor. Based on our findings, these simple bedside tools can help with early prognostication and guide management.

## Introduction

Status epilepticus (SE) is a life-threatening neurological emergency that can lead to substantial morbidity and mortality if not addressed promptly and effectively [1]. The reported annual incidence of SE ranges from 6.8 to 41 per 100,000 individuals, with a bimodal distribution, peaking among children younger than one year and the elderly [2]. The incidence of childhood convulsive SE (CSE) in developed countries is approximately 20 per 100,000 per year, but it varies according to the socioeconomic and ethnic characteristics of the population [3]. The duration of SE is a major determinant of response to antiepileptic therapy and final neurological outcome [4].

The reported mortality rate in SE is 9-21%. CSE results in severe neurological or cognitive sequelae in 11-16% of patients. Therefore, early identification of predictors of poor outcome is essential to guide management and improve prognosis [5]. Bedside neurological assessment tools, such as the Glasgow Coma Scale (GCS) and the Full Outline of UnResponsiveness (FOUR) score, along with seizure duration, are commonly used for prognostication. However, comparative data regarding the predictive utility of these tools remain limited in children. Unlike the GCS, the FOUR score incorporates brainstem reflexes and respiratory patterns and can be applied in intubated patients, making it potentially advantageous in critically ill children. This study, therefore, aimed to assess the role of seizure duration, GCS, and the FOUR score in predicting poor neurological outcomes in pediatric SE, addressing an important gap in the literature.

## Materials and methods

Study design and setting

This was an analytical prospective study carried out at the pediatric ICU of Bundelkhand Medical College, Sagar, Madhya Pradesh, between August 2022 and March 2024. The study was approved by the Institutional Ethics Committee of Bundelkhand Medical College, Sagar, Madhya Pradesh.

Study population

The study included all the patients belonging to the age group of one month to 14 years admitted to the pediatric ICU of Bundelkhand Medical College, Sagar, Madhya Pradesh during the study duration and had a diagnosis of convulsive SE.

Eligibility criteria

Inclusion Criteria

The inclusion criteria were as follows: (a) patients aged between one month and 14 years; (b) patients presenting with a diagnosis of convulsive SE, defined by the International League Against Epilepsy (ILAE) operational definition as any active seizure lasting more than five minutes or recurrent seizures without regaining consciousness between episodes lasting more than five minutes [6]; and (c) patients with pre-existing stable neurodevelopmental conditions, including cerebral palsy.

*Exclusion Criteria* 

The exclusion criteria were as follows: (a) patients aged less than one month or more than 14 years; (b) patients who failed to meet the operational definition of CSE; (c) those who did not provide consent for participation in the study; (d) patients with seizures directly resulting from post-cardiac arrest; (e) patients who were sedated before the initial clinical presentation, precluding accurate baseline GCS or FOUR score assessment; (f) patients with pre-existing severe or profound neurodevelopmental delay or a vegetative state that completely precluded baseline cognitive or motor assessment (outside of stable cerebral palsy); and (g) patients with seizures secondary to acute, severe head trauma (trauma-related seizures).

Sample size

All the eligible children presenting during the study period (August 2022-March 2024) were consecutively enrolled in the study, resulting in a final sample size of 175.

Data collection

A comprehensive history was obtained from a reliable informant after obtaining written informed consent, encompassing the child's demographic details. The clinical profile of children with CSE was evaluated based on seizure duration, GCS, and FOUR scores, and these were fully assessed and documented before the administration of midazolam or other first-line sedatives in our facility and before the use of antiepileptic medications. The baseline GCS and FOUR scores were assessed uniformly during the active convulsive phase. All the information was filled in a predesigned study proforma and safeguarded in a password-protected computer.

The initial GCS score was recorded at presentation, before sedation. It has three components, namely eye opening, verbal response, and motor response [7]. The FOUR score was documented at the time of presentation. It has four components, namely eye response, motor response, brainstem reflexes, and respiration pattern (Table 1) [8].

**Table 1:** Comparison of GCS and FOUR scores GCS: Glasgow Coma Scale; FOUR: Full Outline of UnResponsiveness

Feature	GCS	FOUR score
Eye response	Yes	Yes
Motor response	Yes	Yes
Verbal response	Yes	No
Brainstem reflexes	No	Yes
Respiratory pattern	No	Yes
Applicable in intubated patients	Limited	Yes
Total score range	3-15	0-16

The duration of the seizure was measured from the onset of the clinical seizure (or the last known well time) to the end of the seizure. Seizure duration was classified into two categories (5-30 minutes and > 30 minutes), based on the most recent guidelines from the ILAE, which provide two convulsive SE time points: the time at which continuous seizure activity requiring the administration of emergency medication is likely to be required (t1 = 5 min), and the time at which a continued seizure might lead to permanent neuronal damage (t2 = 30 min).

Outcome

Patient outcomes at hospital discharge were initially classified into three distinct clinical categories: (a) death, which was defined as in-hospital mortality; (b) neurological deficit, which was defined as any new-onset, objective motor, cognitive, or behavioral impairment not present before the index episode of SE (accounting for baseline status in patients with pre-existing cerebral palsy); and (c) recovery, which was defined as a complete return to the patient's baseline neurological status without any new-onset deficits.

Neurological deficits were clinically evaluated through standardized physical and neurological examinations at the time of hospital discharge. To fulfill the requirements for multivariable logistic regression analysis, these categorical outcomes were collapsed into a binary dependent variable (poor outcome versus good outcome). Specifically, "poor neurological outcome" was defined strictly as in-hospital mortality (coded as 1; n = 15 events). "Good outcome" was defined as overall survival, which included both patients who completely recovered and those discharged with a neurological deficit (coded as 0).

Statistical analysis

Data were entered into Microsoft Excel and analyzed using SPSS Statistics version 31 (IBM Corp., Armonk, NY). Categorical and nominal variables, including patient sex, primary diagnosis, and seizure duration categories, were expressed as frequencies and percentages. Baseline categorical data comparisons across neurological outcome groups were evaluated using the Chi-square test. Continuous clinical variables, specifically the initial GCS and FOUR scores, were expressed as mean and standard deviation (SD) along with their respective minimum and maximum ranges. Baseline comparisons of these continuous clinical scores across the three outcome categories (discharge, neurological deficit, and death) were analyzed using one-way analysis of variance (ANOVA) based on normality assumptions.

To identify clinical predictors of a poor neurological outcome (defined as a composite of neurological deficit or death), logistic regression models were constructed. Univariate logistic regression was first performed to calculate unadjusted odds ratios (OR) and 95% confidence intervals (CI) for individual clinical parameters (GCS score, FOUR score, seizure duration, sex). Variables were subsequently entered into a multivariate logistic regression model to calculate adjusted odds ratios (AOR) and control for potential confounding factors. All statistical tests were two-tailed, and a p-value < 0.05 was considered statistically significant.

## Results

A total of 175 children with SE were included in the study. As shown in Table 2, the majority were in the age group of one to five years (n = 77, 44.0%), with a mean age of 3.9 ± 3.5 years. Males constituted the majority (n = 107, 61.1%), compared to females (n = 68, 38.9%). Most patients recovered without neurological sequelae (n = 152, 86.9%), while eight (4.6%) experienced neurological deficits and 15 (8.6%) died. Seizure duration ranged from 5-30 minutes in 150 patients (85.7%), whereas it exceeded 30 minutes in 25 patients (14.3%). The mean FOUR score was 12.2 ± 2.014 (range: 2-16), while the mean GCS score was 10.7 ± 1.811 (range: 3-15) (Table 3).

**Table 2:** Characteristics of the study population

Variable	Category	N (%)
Age	Less than 2 months	08 (4.6)
	2 months–1 year	33 (18.9)
	1–5 years	77 (44.0)
	> 5 years	57 (32.5)
Outcome	Recovery	152 (86.9)
	Neurological deficit	08 (4.6)
	Death	15 (8.6)
Sex	Male	107 (61.1)
	Female	68 (38.9)
Seizure duration	5–30 minutes	150 (85.7)
	> 30 minutes	25 (14.3)

**Table 3:** Clinical scores: descriptive data SD: standard deviation; FOUR: Full Outline of UnResponsiveness; GCS: Glasgow Coma Scale

Variable	N	Mean ± SD	Minimum	Maximum
FOUR score	175	12.20 ± 2.01	2	16
GCS score	175	10.70 ± 1.81	3	15

Children with seizure duration > 30 minutes had markedly higher mortality (56%) compared to those with seizure duration between 5-30 minutes (0.7%). As shown in Table 4, comparison of mean GCS and FOUR scores across outcome groups showed significant differences (GCS: F = 19.84, p < 0.001; FOUR: F = 22.67, p < 0.001). Patients who recovered had significantly higher mean scores, whereas the lowest scores were observed in children who died. Febrile seizures were the most common etiology (n = 53, 30.3%), followed by central nervous system infections (n = 48, 27.4%) and seizure disorders (n = 34, 19.4%). Other etiologies included hypoxic seizures (n = 15, 8.6%), cerebral palsy (n = 8, 4.6%), hypoxic-ischemic encephalopathy sequelae (n = 7, 4%), metabolic disorders (n = 2, 1.1%), and isolated cases of hepatic encephalopathy, cyanotic heart disease, hypertensive encephalopathy, and organophosphate poisoning (Table 5).

**Table 4:** Comparison of mean GCS and FOUR scores across outcomes SD: standard deviation; FOUR: Full Outline of UnResponsiveness; GCS: Glasgow Coma Scale

Variable	Outcome	Mean ± SD
GCS score	Recovery	10.96 ± 1.61
	Neurological deficit	10.25 ± 1.98
	Death	8.33 ± 1.95
FOUR score	Recovery	12.51 ± 1.61
	Neurological deficit	11.00 ± 3.85
	Death	9.73 ± 2.54

**Table 5:** Distribution of diagnoses (N = 175)

Diagnosis	N	%
Febrile seizures	53	30.3
Central nervous system infection	48	27.4
Seizure disorder	34	19.4
Hypocalcaemic seizures	04	2.3
Hypoxic seizure	15	8.6
Hepatic encephalopathy	01	0.6
Cyanotic heart disease	01	0.6
Hypertensive encephalopathy	01	0.6
Metabolic disorder	02	1.1
Hypoxic ischemic encephalopathy sequelae	07	4.0
Cerebral palsy	08	4.6
Organophosphate poisoning	01	0.6

As presented in Table 6, on univariate logistic regression analysis, lower GCS score (OR = 0.53, 95% CI: 0.40-0.71; p < 0.01), lower FOUR score (OR = 0.56, 95% CI: 0.42-0.74; p = 0.004), and seizure duration >30 minutes (OR = 26.63, 95% CI: 9.12-77.72; p < 0.001) were significantly associated with poor neurological outcome. Sex was not significantly associated with neurological outcome (p = 0.856, chi-square value = 0.033). However, seizure duration >30 minutes and refractory status epilepticus were strongly associated with poor neurological outcomes (p < 0.01) (Table 7).

**Table 6:** Univariate logistic regression for poor neurological outcomes GCS: Glasgow Coma Scale; FOUR: Full Outline of UnResponsiveness; CI: confidence interval

Variables	B	S.E.	Wald	Sig. (p-value)	Unadjusted odds ratio	95% CI	
						Lower limit	Upper limit
GCS score	-0.63	0.15	17.95	0.0012	0.53	0.40	0.71
FOUR score	-0.58	0.14	16.15	0.004	0.56	0.42	0.74
Seizure duration > 30 min	3.28	0.55	36.00	< 0.001	26.63	9.12	77.72
Sex (male)	-0.22	0.45	0.24	0.625	0.80	0.33	1.95

**Table 7:** Association between categorical variables and neurological outcomes

Variable	Category	Recovery	Poor outcome	Total	Chi-square	P-value
Sex	Male	97 (90.7)	10 (9.3)	107	0.033	0.86
	Female	63 (92.6)	05 (7.4)	68		
Duration of seizure	≤ 30 minutes	149 (99.3)	01 (0.7)	150	76.809	< 0.01
	> 30 minutes	11 (44.0)	14 (56)	25		
Refractory status epilepticus	Yes	17 (54.8)	14 (45.2)	31	58.812	< 0.01
	No	143 (99.3)	01 (0.7)	144		

A statistically significant association was observed between seizure duration and neurological outcome (χ² = 83.938, p < 0.001). (Table 8). As shown in Table 9, the final multivariable logistic regression model included four predictor variables (GCS, FOUR score, sex, and seizure duration) and 15 patients with poor neurological outcomes. On multivariate logistic regression analysis, only seizure duration > 30 minutes remained a significant independent predictor of poor neurological outcome (AOR = 12.14, 95% CI: 3.14-43.26; p < 0.001), whereas GCS and FOUR scores lost statistical significance after adjustment.

**Table 8:** Association between seizure duration and neurological outcomes Chi-square: 83.938; p-value: < 0.01

Seizure duration	Discharge, n (%)	Deficit, n (%)	Death, n (%)	Total
5–30 minutes	142 (94.7%)	7 (4.7%)	1 (0.7%)	150
> 30 minutes	10 (40.0%)	1 (4.0%)	14 (56.0%)	25

**Table 9:** Multivariate logistic regression for poor neurological outcomes GCS and FOUR are entered into univariate and multivariate logistic regression models as continuous variables and were not dichotomized using pre-defined cut-off values GCS: Glasgow Coma Scale; FOUR: Full Outline of UnResponsiveness; CI: confidence interval

Variables	B	S.E.	Wald	Sig. (p-value)	Adjusted odds ratio	95% CI	
						Lower limit	Upper limit
GCS score	-0.19	0.21	0.78	0.377	0.83	0.54	1.26
FOUR score	-0.19	0.19	0.99	0.319	0.83	0.57	1.20
Seizure duration > 30 min	2.49	0.64	14.82	< 0.001	12.14	3.14	43.26
Sex (male)	-0.03	0.57	0.003	0.952	0.97	0.31	2.97

## Discussion

SE is a life-threatening neurological emergency in children, associated with significant morbidity and mortality. Hence, it is necessary to devise simple bedside tools and determine prognosis-determining factors to guide management and improve neurological outcome. In our cohort, the majority of children were aged between one and five years (n = 77, 44.0%), followed by those aged more than five years (n = 57, 32.5%), two months to one year (n = 33, 18.8%), and less than two months (n = 8, 4.5%). The mean age of the study participants was 3.9 ± 3.5 years. Das et al. [9] and Kumar et al. [10] also observed similar results. The increased frequency of SE in young children reflects both the increased susceptibility of the immature brain to seizures and the common occurrence of CNS infections such as meningitis and encephalitis in young children.

In this study, male children outnumbered females, with 107 (61.1%) males and 68 (38.9%) females. A similar male predominance was reported by Bakolia et al. [11], whereas Ajmariya et al. [12] reported a nearly equal sex distribution. Regarding outcomes, 152 of the 175 participants (86.9%) recovered completely, eight (4.6%) developed neurological deficits at discharge, and 15 (8.6%) died. These findings are comparable to those of Amonkar et al. [13], who reported a mortality rate of 8.9%. In contrast, several studies from developing countries have documented higher mortality rates. For example, Uzair M et al. [14] reported that eight of the 16 deaths (50.0%) were attributed to acute symptomatic etiology. With respect to seizure duration, 150 patients (85.7%) had seizures lasting 5-30 minutes, while 25 (14.3%) experienced seizures lasting more than 30 minutes. By comparison, Dodiyar et al. [15] reported seizure durations of 5-30 minutes in 38 (36.2%) cases, 30-60 minutes in 62 (59.1%) cases, and more than 60 minutes in five (4.8%) cases out of a total of 105 patients.

We found that lower initial GCS and FOUR scores were associated with poor outcomes on univariate analysis, but only prolonged seizure duration remained an independent predictor on multivariate analysis. Children with lower GCS scores at presentation had a higher risk of death. Similar findings were reported in previous studies that have established GCS as a reliable predictor of neurological outcome in critically ill pediatric patients [16-17]. This is because a lower GCS score reflects more severe cortical dysfunction and underlying brain injury, which contribute to poor neurological outcomes. Similarly, the FOUR score showed a strong association with adverse outcomes on univariate analysis. Unlike the GCS, the FOUR score can be used in intubated patients as it considers brainstem reflexes and respiratory patterns instead of the verbal response. Previous studies have suggested that the FOUR score may have superior prognostic accuracy compared with the GCS in intensive care settings [18-19].

According to the ILAE 2015 definition of CSE, seizure activity lasting ≥ 5 minutes (t1) warrants emergency treatment, whereas seizure duration beyond 30 minutes (t2) is associated with an increased risk of neuronal injury and adverse neurological outcomes [6]. Therefore, seizure duration was categorized as 5-30 minutes and > 30 minutes. This finding may reflect the overlap in neurological information captured by both scales and the strong prognostic effect of seizure duration. Prolonged seizures are directly associated with neuronal injury and may therefore exert a greater influence on outcome than baseline neurological assessment scores.

Although lower GCS and FOUR scores were significantly associated with mortality on univariate analysis, neither remained independently significant after adjustment; only seizure duration remained the independent significant predictor of outcome on multivariate analysis. The GCS and FOUR score assess related neurological domains; some overlap in prognostic information is expected. This also explains why both variables lost significance after adjustment, whereas seizure duration remained independently associated with outcome on multivariate analysis. Patients with seizures lasting more than 30 minutes had an increased risk of death and neurological deficits. This finding aligns with existing evidence that prolonged seizures lead to excitotoxic neuronal injury, mitochondrial dysfunction, and systemic complications [5].

Assessment of GCS, FOUR score, along with seizure duration, provides an effective approach for predicting outcome in pediatric SE. These tools are simple, bedside, and can be rapidly assessed without the need for advanced investigations, making them particularly valuable in resource-limited settings. Their use may help treating physicians identify high-risk patients and monitor them closely with early escalation of therapy and closer follow-up. The study also found that gender was not significantly associated with neurological outcomes. Common etiologies observed were febrile seizures and CNS infections. Previous studies have shown that acute symptomatic causes, particularly CNS infections and hypoxic insults, are associated with poorer outcomes compared to febrile or idiopathic seizures [20-21].

Limitations

This study has several limitations. First, it was conducted at a single tertiary-care center, which may limit the generalizability of the findings. Second, neurological outcomes were assessed only until hospital discharge, and long-term neurodevelopmental follow-up data were unavailable. Third, important potential confounders, including etiology, time to treatment, need for mechanical ventilation, and treatment response, were not fully incorporated into the multivariable model because only 15 mortality events were observed. To minimize the risk of model overfitting, additional covariates were not included in the multivariable analysis. Consequently, residual confounding cannot be excluded, and adjustment for pre-hospital treatment and time to presentation was also not performed. Furthermore, the statistical model was limited by the inherent multicollinearity between the GCS and FOUR score. Because both scales assess overlapping neurological domains and share substantial variance, their simultaneous inclusion in the multivariable model may have inflated standard errors, potentially explaining why neither score remained statistically significant after adjustment for seizure duration. Finally, the relatively small number of patients with neurological deficits limited subgroup analyses, and mortality alone was used as the primary adverse outcome for logistic regression. Future studies should include receiver operating characteristic (ROC) curve analysis to compare the diagnostic performance of the GCS and FOUR score and perform a formal assessment of multicollinearity between these two scoring systems.

## Conclusions

In our cohort, lower GCS and lower FOUR scores, along with seizure duration, were significantly associated with poor neurological outcomes on univariate analysis. Despite this, both scoring systems remain useful bedside instruments for early risk stratification. Specifically, a seizure duration greater than 30 minutes emerged as the only independent predictor of poor neurological outcome (death) on multivariate analysis.
